# Postnatal onset of retinal degeneration by loss of embryonic *Ezh2* repression of Six1

**DOI:** 10.1038/srep33887

**Published:** 2016-09-28

**Authors:** Naihong Yan, Lin Cheng, Kinsang Cho, Muhammad Taimur A. Malik, Lirong Xiao, Chenying Guo, Honghua Yu, Ruilin Zhu, Rajesh C. Rao, Dong Feng Chen

**Affiliations:** 1 Department of Ophthalmology and Ophthalmic Laboratories, State Key Laboratory of Biotherapy and Cancer Center, West China Hospital, Sichuan University, Chengdu, Sichuan, P. R. China; 2 Schepens Eye Research Institute, Massachusetts Eye & Ear, Department of Ophthalmology, Harvard Medical School, Boston, Massachusetts, USA; 3 State Key Laboratory of Ophthalmology, Zhongshan Ophthalmic Center, Sun Yat-Sen University, 54 South Xianlie Road, Guangzhou, Guangdong, P. R. China; 4 Department of Ophthalmology and Visual Sciences, W. K. Kellogg Eye Center, Comprehensive Cancer Center, Department of Pathology, University of Michigan Ann Arbor, Michigan, USA; 5 Division of Ophthalmology, Surgical Service, Veterans Administration Ann Arbor Healthcare System, Ann Arbor, Michigan, USA; 6 Veterans Administration Boston Healthcare System, Boston, Massachusetts, USA

## Abstract

Some adult-onset disorders may be linked to dysregulated embryonic development, yet the mechanisms underlying this association remain poorly understood. Congenital retinal degenerative diseases are blinding disorders characterized by postnatal degeneration of photoreceptors, and affect nearly 2 million individuals worldwide, but ∼50% do not have a known mutation, implicating contributions of epigenetic factors. We found that embryonic deletion of the histone methyltransferase (HMT) *Ezh2* from all retinal progenitors resulted in progressive photoreceptor degeneration throughout postnatal life, via derepression of fetal expression of *Six1* and its targets. Forced expression of *Six1* in the postnatal retina was sufficient to induce photoreceptor degeneration. *Ezh2*, although enriched in the embryonic retina, was not present in the mature retina; these data reveal an *Ezh2*-mediated feed-forward pathway that is required for maintaining photoreceptor homeostasis in the adult and suggest novel targets for retinal degeneration therapy.

Postnatal degeneration of photoreceptors underlies the most common causes of irreversible blindness. Rod-cone photoreceptor dystrophy is a group of congenital retinal degenerative disorders characterized by postnatal onset of progressive photoreceptor cell loss^1^,^2^, leading to severe visual impairment and deterioration. Molecular genetic studies have revealed the underlying causes in only ∼50% of these patients. Striking characteristics of these diseases are their genetic heterogeneity and large variability in the age of onset, progression, and final visual outcome that can be found even between monozygotic twins^3^,^4^,^5^, suggesting the impact of factors other than genetics on its pathogenesis. Contribution of epigenetically-regulated events *in utero* toward the adult onset of retinal degeneration is unclear.

Due to minimal cell turnover, the postnatal retina is particularly vulnerable to insults during embryonic development. Epigenetic events mediated by repressive histone methylation regulate the transcriptional programs in proliferating embryonic stem cells and their differentiated, post-mitotic progeny by suppressing transcription in specific fetal genes. These early developmental events may serve as a nexus point of cell proliferation and differentiation that persists into adulthood to allow establishment of stabilized expression of postnatal genes and maintain tissue homeostasis. Perturbation of this process, via inhibition of repressive histone methyltransferase (HMT) activity in embryonic retinal progenitors, may lead to disruption of postnatal homeostasis. Consequently, inhibition of repressive HMT activity could lead to postnatal retinal degeneration through “developmental reprogramming” that stems from aberrant derepression of a subset of normally silenced genes^6^.

A candidate repressive HMT whose embryonic disruption may interfere with postnatal retinal homeostasis is *enhancer of zeste homolog 2* (*Ezh2*), the enzymatic subunit of the polycomb repressive complex 2 (PRC2). Ezh2 trimethylates histone H3 at lysine 27 (H3K27me3), a repressive histone mark that is known to persist into adulthood^7^,^8^. Previous reports have shown that *Ezh2*/*EZH2* is abundant during embryonic development but is absent during the late postnatal period and adulthood in mice and humans^9^,^10^,^11^. Conditional loss-of-function experiments in which an early (E9.5) retinal Cre driver, such *as Dkk3*-12 or *Pax6-αCre*, was used to delete retinal *Ezh2,* has demonstrated a role for this HMT in the proliferation and differentiation of retinal progenitor cells (RPCs)^13^,^14^. To circumvent this issue and focus on a role for *Ezh2*-mediated repressive modifications in postnatal retinal homeostasis, we generated mice carrying *Ezh2* deficiency selectively in RPCs at a later time point (E11.5 and later), using *Chx10* promoter^15^,^16^,^17^. Here we report that *Chx10-Cre; Ezh2* deficient retinae developed normally to the perinatal period; however *Ezh2* mutant retinae underwent progressive photoreceptor degeneration postnatally, a period during which *Ezh2* is no longer expressed in wild type retinae. Taken together, these findings provide new insights into how dysregulation of *Ezh2* function in development may contribute to retinal degeneration in postnatal life.

## Results

### *Ezh2* deficiency driven by *Chx10-Cre* induces progressive retinal degeneration in postnatal life

**Figure 1:**
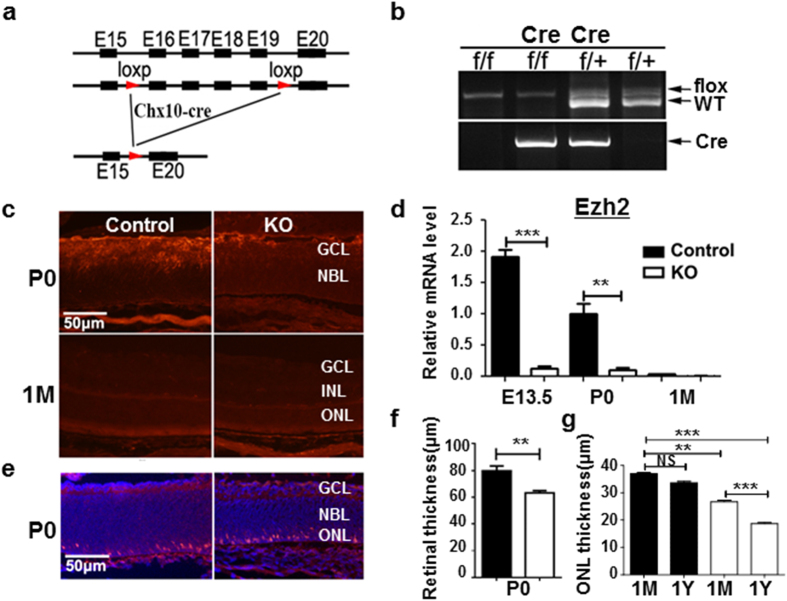
*Ezh2* deletion induces progressive retinal degeneration. (**a**) Intron/exon diagram of mice *Ezh2* and *Ezh2*^*flox/flox*^ alleles. (**b**) Genotyping of *Ezh2* and *Chx10-cre* genes. (**c,d**) Immunohistochemistry (**c**) and qPCR (**d**) assessment of *Ezh2* expression in the retinas of control and KO mice: note that *Ezh2* expression was detected in the P0 control retina, but was lost in the adult retina. (**e**) Retinal sections labeled for nuclear marker DAPI (blue) and photoreceptor cell marker anti-Recoverin (red). (**f**) Retinal thickness measured in P0 retinal sections. (**g**) Quantification of ONL thickness in retinal sections of mice at different ages, showing progressive ONL degeneration from 1 M to 1 Y in the KO mouse retina. Bars represent the mean ± S.D. of at least six biological replicates. NS indicates no significant difference; **indicates P < 0.01; ***indicates P < 0.001.

Mice with loxP sites flanking exons encoding the catalytic SET domain of *Ezh2* (*Ezh2*^*flox/flox*^) (Fig. 1a) were crossed with mice expressing Cre-recombinase under the control of a bacterial artificial chromosome encoding *Chx10* (*Chx10-Cre*). The *Chx10-cre* mouse line directs high-level Cre expression after E11.5 onward in intermediate and late RPCs, which leads to retinal-specific excision in virtually all RPC-derived cells of the retina^16^. All mice were genotyped by PCR of tail genomic DNA for *Ezh2*^*flox/flox*^ alleles (Fig. 1b). In knockout (KO or *Chx10-cre;Ezh2*^*flox/flox*^) mice, the level of *Ezh2* expression was nearly undetectable at E13 through postnatal 1 month (1 M) of age (Fig. 1c,d). Consequently, *H3K27me3* deposition in retinal progenitor cells (RPCs; neuroblast layer) of KO mice was largely decreased compared to WT controls at E13.5 (Supplementary Fig. 1). In contrast, *Ezh2* was detected in RPCs of the embryonic retinas of control mice (*Ezh2*^*flox/flox*^); at P0, this expression was limited to the ganglion cell layer (GCL; Fig. 1c) but its expression was extinguished in the retina at 1 M (Fig. 1c,d). These data confirmed that *Ezh2* was successfully deleted in the retina of KO mice.

To assess the effect of *Ezh2* deletion, we compared the morphology and numbers of retinal neurons in KO mice to control mice at different stages. KO mice exhibited largely normal structured retina at birth; however, despite the normally absent expression of *Ezh2* at 1 M, KO retina underwent progressive degeneration that gradually occurred from 1 M throughout adulthood. Consistent with the reported role of *Ezh2* in regulating RPC proliferation and retinogenesis, the retina of KO mice (Fig. 1e,f) was slightly (∼20%) thinner than their littermate controls or those with heterozygous *Ezh2* deletion (*Ezh2*^+/−^ or *Chx10-cre; Ezh2*^*flox*/+^) at P0.

**Figure 2:**
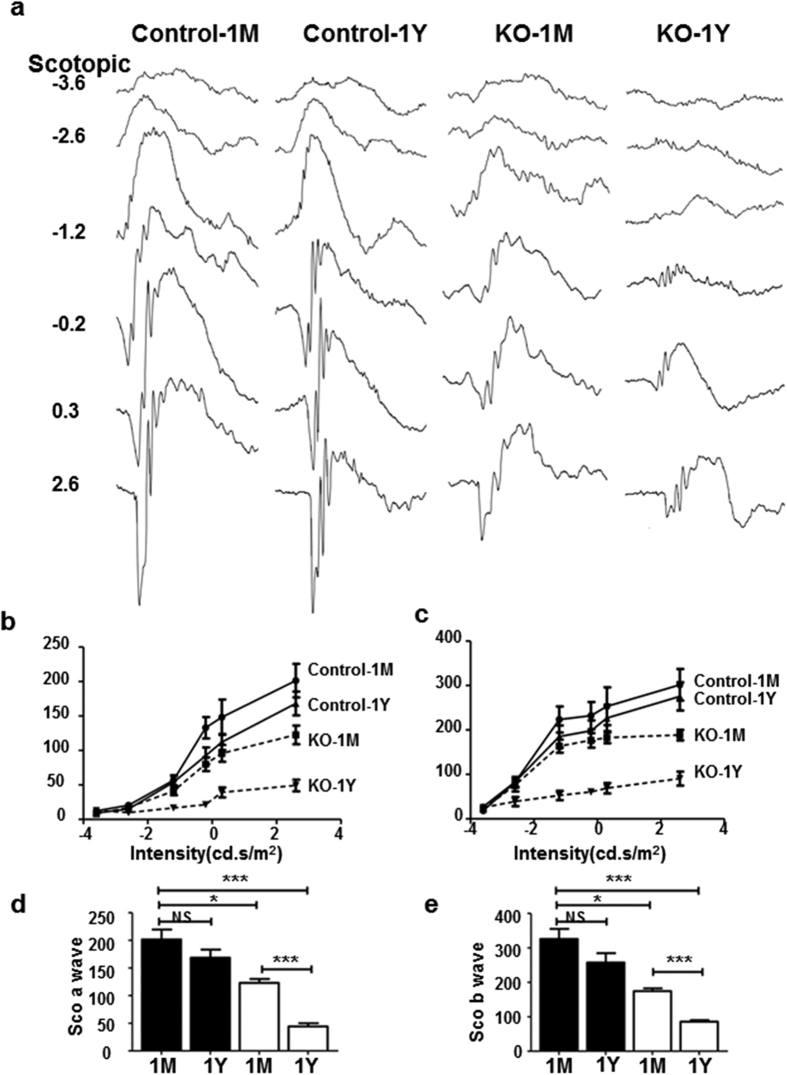
*Ezh2* deletion impairs postnatal photoreceptor cell function. (**a**) Representative ERG waveforms of control and KO mice that were subjected to flashes of increasing intensities under scotopic conditions, which isolate rod photoreceptor-mediated responses. (**b–e**) The amplitudes of scotopic ERG a- and b-waves, which measures cone responses: Black bar, WT controls; white bar, KO. Note the abnormal and progressive reduction of ERG a- and b-wave amplitudes in KO mice from 1 M to 1 Y. Data represent the mean ± S.D. of at least six biological replicates. NS indicates no significant difference; *indicates P < 0.05; **indicates P < 0.01; ***indicates P < 0.001.

The thickness of the retina, especially the outer nuclear layer (ONL), continued to decrease from 1 M to 12 M (Fig. 1g and Supplementary Fig. 2). This was confirmed by measuring retinal thicknesses in both H&E stained retinal sections (Supplementary Fig. 2a,b) and by non-invasive imaging with high-resolution spectrum domain-optic coherence topography in live animals (Supplementary Fig. 2d,e). The ONL thickness of 1 M KO mice was ∼25% thinner than the control mice; by 12 M, and it was reduced to 50% of that in littermate controls. Ultrastructural analysis of photoreceptor outer segments (OS) with electron microscopy revealed less dense and sometimes disintegrated OS in 1 M KO mice, supporting the on-going photoreceptor degeneration in these mice; whereas, the retina of their littermate controls exhibited well-aligned OS discs (Supplementary Fig. 2f). Moreover, electroretinography (ERG) analyses showed progressive loss of photoreceptor functions as measured by scotopic a-wave and photopic b-wave, which represent rod and cone functions, respectively. From 1 M to 12 M, the amplitudes of scotopic a-wave of KO mice reduced from 65% of that of littermate controls to 20%; scotopic (Fig. 2e) and photopic (not shown) b-waves exhibited similar reduction from 1 M to 12 M (Fig. 2). Less robust, but significant, loss of bipolar cells (10% at 1 M and 25% at 12 M) was also observed in KO retinae as compared with that of littermate controls (Supplementary Fig. 3). No significant loss of RGCs was noted in the KO retina as compared to littermate control mice (not shown). Together, our data indicates that *Ezh2* deficiency during development results in progressive retinal, primarily photoreceptor, degeneration throughout the postnatal period.

### *Ezh2* deficiency results in selective dysregulation of *Six1* and photoreceptor-related genes

**Figure 3:**
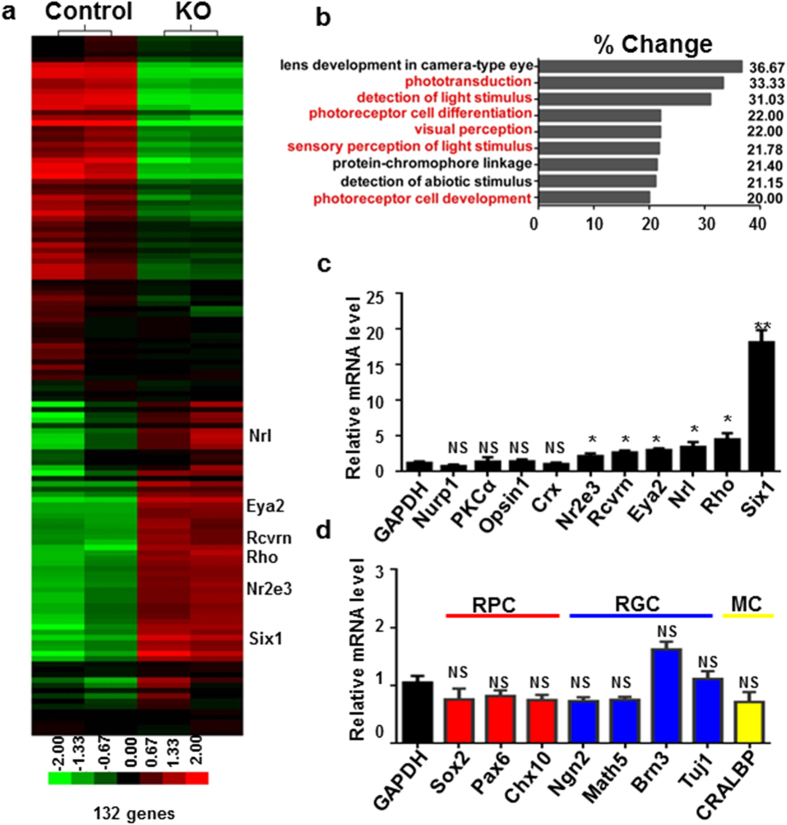
*Ezh2* deletion promotes derepression of *Six1* and photoreceptor-related genes. (**a**) Heat map of clustered microarray data from P0 retina showed 132 genes changed >1.5 fold between control and KO mice. (**b**) GO analysis of functional categories identified a high number of genes that are directly involved in photoreceptor development, differentiation and photosensitivity. (**c,d**) qPCR results from P0 WT and KO retina showed the relative expression of retinal progenitor cell (RPC) and neuron-related genes in KO mice at P0. RGCs, retinal ganglion cells; MC, Müller cells. Note the significant elevation of *Six1*, *Eya2* and rod photoreceptor-related gene expression, including *Nr2e3*, *Recoverin* (Rcvrn), *Nrl* and *Rhodopsin* (Rho), in KO mice. Bars represent the mean ± S.D. of at least six biological replicates. NS indicates no significant difference; *indicates P < 0.05; **indicates P < 0.01; ***indicates P < 0.001.

To define the genetic landscape of the KO vs control retinae, we analyzed gene transcripts in the P0 retina using Affymetrix cDNA microarray. P0 was selected because this is when *Ezh2* disappears from the outer neuroblast layer or RPCs while KO retina revealed a largely normal appearance without massive degeneration. Surprisingly, instead of finding a broad change in gene expression profiles, our comparison of over 24,000 cDNA transcripts (detected from >28,000 transcripts being screened) of KO vs. control retinae of P0 mice revealed only 132 genes with over 1.5 fold change (Fig. 3a and Table 1). As *Ezh2* is usually considered to deposit a repressive mark, we performed gene Ontology (GO) (http://david.abcc.ncifcrf.gov/) analysis^18^,^19^ particularly for genes that were upregulated in KO mice and likely repressive targets of *Ezh2*. Remarkably, the large majority of these genes were related to photoreceptor development or function (Fig. 3b,c), implicating selective and cell type-specific gene regulation or suppression in RPCs by *Ezh2*.

We did not observe changes in the expression of genes related specifically to other retinal cell types, including RPCs, RGCs, bipolar cells, and Müller cells (Fig. 3d). Results of qPCR analysis in P0 retina confirmed the microarray data, and showed specific upregulation of photoreceptor related genes, such as *Nrl*, *Nr2e3*, *recoverin* and *rhodopsin*; but not genes related to RPCs (e.g. *Sox2* and *Pax6*), RGCs (e.g., *Ngn2, Math5, Brn3a,* and *Tuj1*) and other retinal cell types (e.g. *PKCα* and *CRALBP*) (Fig. 3c,d). Along with photoreceptor degeneration in the KO retina at 1 M, the levels of expression of photoreceptor related genes were also downregulated while the expression of other non-photoreceptor-related genes remained unchanged (not shown). Despite the upregulation of *Lin28b* and *Eya2* – markers of RPCs for early born neurons (e.g. RGCs) – no significant changes in numbers of RGCs or levels of expression of RGC related genes were noted in the postnatal retinae of KO mice. The increase of photoreceptor specific protein expression in KO mice was consistent with the previous report^14^ and was further verified by immunofluorescence labeling. Moderate elevation of Recoverin- and Rhodopsin-immunofluorescence intensities was detected in P3 KO retinae compared to littermate WT controls (Supplementary Fig. 4). The data suggest selective destabilization of photoreceptor gene expression associated with photoreceptor degeneration in the KO retina.

### Targeted deletion of *Ezh2* from RGCs does not affect cell homeostasis or RGC gene expression

**Figure 4:**
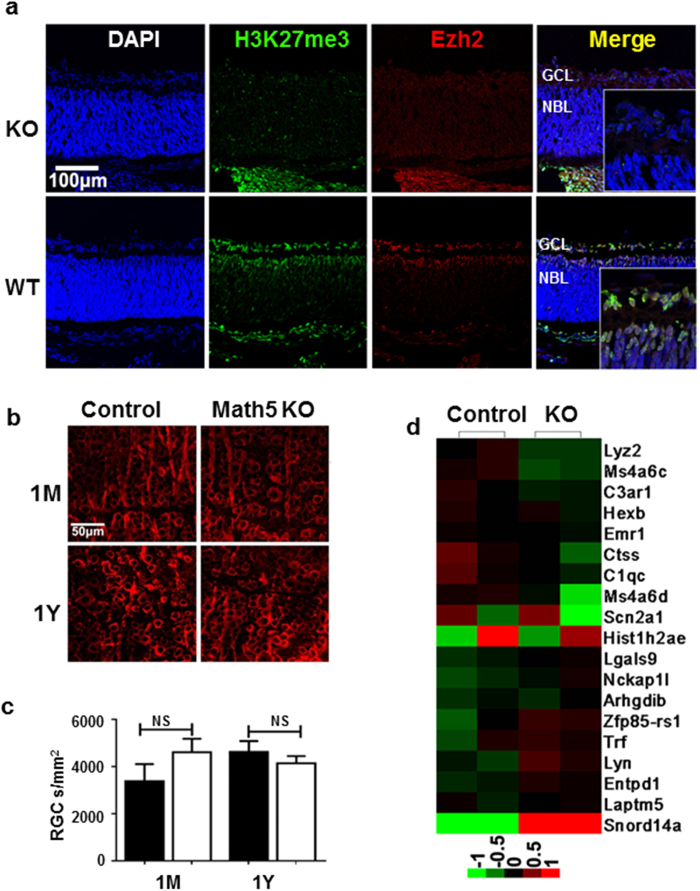
RGC-specific *Ezh2* inactivation reduces *H3K27me3* deposition and gene expression of selective transcripts. (**a**) Absence of H3K27me3 deposition (green) and *Ezh2* expression (red) in the GCL of P0 *Math5-KO* retina compared to P0 control (*Ezh2*^*flox/flox*^) mouse retina. (**b,c**) Assessment of RGC (red) morphology (**b**) and cell counts (**c**) in Tuj1-immunolabeled retinal flat-mounts of adult control Black bar: WT controls; white bar: Math5-KO. (*Ezh2*^*flox/flox*^) and *Math5-KO* mice showed no significant differences in RGC morphology or cell number. (**d**) Heat map of microarray analysis using RNAs collected from RGCs purified in P0 *Math5-KO* and littermate controls. One gene was up-regulated and 18 genes were down-regulated over 1.5 fold in *Math5-KO* mice compared to control mice. Bars represent the mean ± S.D. of at least six biological replicates.

The finding that RPC inactivation of *Ezh2* induces primarily photoreceptor degeneration and gene expression changes suggests that *Ezh2* selectively regulates photoreceptor related genes. If this hypothesis is true, *Ezh2* deficiency in other retinal cell types should not directly cause retinal neurodegeneration. To test this hypothesis, we conditionally inactivated *Ezh2* from all RGC progenitors, using a *Math5-Cre* driver (*Math5-*KO or *Math5-Cre*^+^; *Ezh2*^*flox/flox*^)^20^ which mediates gene excision from RPCs that are competent to generate RGCs from E11.5 onward. *Ezh2* expression and *H3K27me3* deposition in the outer neuroblastic layer of *Math5*-KO mice was maintained, but was abolished in the GCL of embryonic and P0 mice, as expected. In *Math5*-KO mice, we observed no RGC loss, nor morphological or functional defects throughout the adulthood (Fig. 4a–c). We next conducted an Affymetrix cDNA microarray analysis to compare gene transcripts in purified RGCs of P0 WT and *Math5*-KO mice. In support of the above findings that deletion of *Ezh2* from intermediate or late stage RPCs selectively impact the expression of photoreceptor related genes, only a few changes were detected when compared to littermate controls (*Ezh2*^*flox/flox*^) (Fig. 4d). Gene Ontology analysis showed no specific association of these gene expression changes with RGCs or RPCs (Supplementary Fig. 5). Thus, disruption of *Ezh2*-mediated disposition of repressive marks selectively affects the gene expression and homeostasis of photoreceptor cells in the postnatal mouse retina.

### Forced expression of *Six1* in the postnatal retina induces photoreceptor degeneration

**Figure 5:**
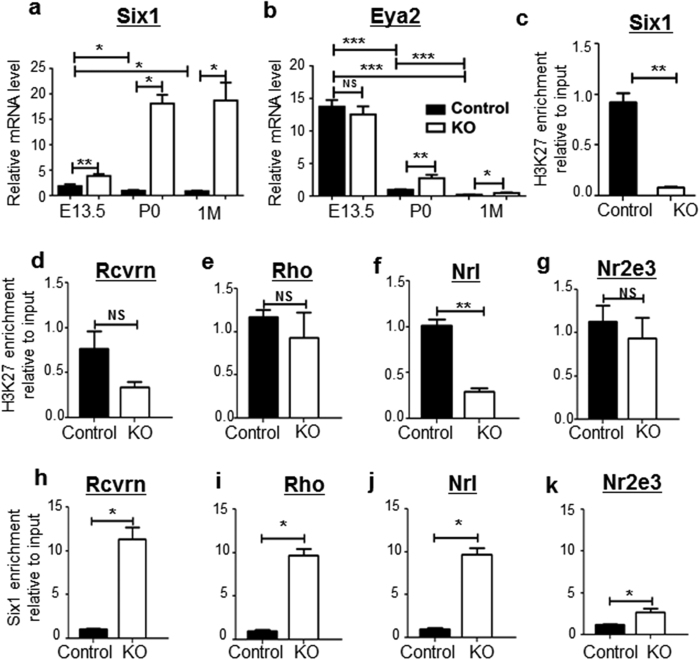
*Ezh2* deficiency causes derepression of *Six1* and dysregulation of photoreceptor gene expression in the postnatal retina. (**a,b**) qPCR results showed aberrantly increased expression of *Six1* and *Eya2* genes in the postnatal retina of KO mice. (**c–g**) DNA ChIP analysis showed significant reductions of *Ezh2* binding to *Six1* and *Nrl* promoters, but not to *Recoverin*, *Rhodopsin* or *Nr2e3* promoters, in P0 KO retinas compared to littermate control retinas, suggesting that *Ezh2* directly targets *Six1* and *Nrl.* (**h–k**) KO retinae exhibited significantly increased enrichment of *Six1* binding to the promoters of photoreceptor-related genes, *Recoverin (Rcvrn)*, *Rhodopsin (Rho)*, *Nrl* and *Nr2e3* genes. Bars represent the mean ± S.D. of at least six biological replicates. NS indicates no significant difference; **indicates P < 0.01.

*Ezh2* mediates gene repression via catalyzing H3K27me3 on its target genes. We proposed that embryonic loss of *Ezh2* resulted in aberrant sustained expression of its target genes in the postnatal period, which are normally “switched off” during the perinatal period, leading to destabilized photoreceptor gene expression and degeneration throughout the perinatal and postnatal periods. We found that the homeodomain protein *Six1*, which was detected in the embryonic retina, was aberrantly upregulated—from nearly 4 to 20-fold—at E13.5, P0, and 1 M, in KO vs littermate control retinae (Fig. 5a). *Eya2*, a co-factor of *Six1*, followed a similar pattern and was also upregulated at P0 and 1 M of KO retina (Fig. 5b). We next asked if *Ezh2* directly deposited the repressive mark H3K27me3 onto *Six1* by performing chromatin-immunoprecipitation (ChIP) assay with H3K27me3 antibody. *Ezh2* deficiency resulted in *H3K27me3* depletion from the *Six1* and *Nrl* promoters, but not photoreceptor genes, that include *Recoverin*, *Rhodopsin*, and *Nr2e3* (Fig. 5c–g). These results suggest that *Six1*, but not the majority of photoreceptor genes, is a direct target of *Ezh2*. We next performed the ChIP assay to determine if *Six1* binds the core promoters of photoreceptor genes to regulate their expression. We noted that in KO mice, *Six1* occupancy was differentially enriched at the *Recoverin*, *Rhodopsin, Nrl* and *Nr2e3* promoters compared to controls (Fig. 5h–k), indicating that *Six1* regulates the expression of photoreceptor-specific genes. These results suggest the following: upon loss of *Ezh2* and H3K27me3, aberrantly expressed *Six1*, which binds *Eya2* to mediate transcriptional activation, contributes to abnormally elevated expression of photoreceptor genes at P0, prior to their downregulation following postnatal photoreceptor degeneration.

**Figure 6:**
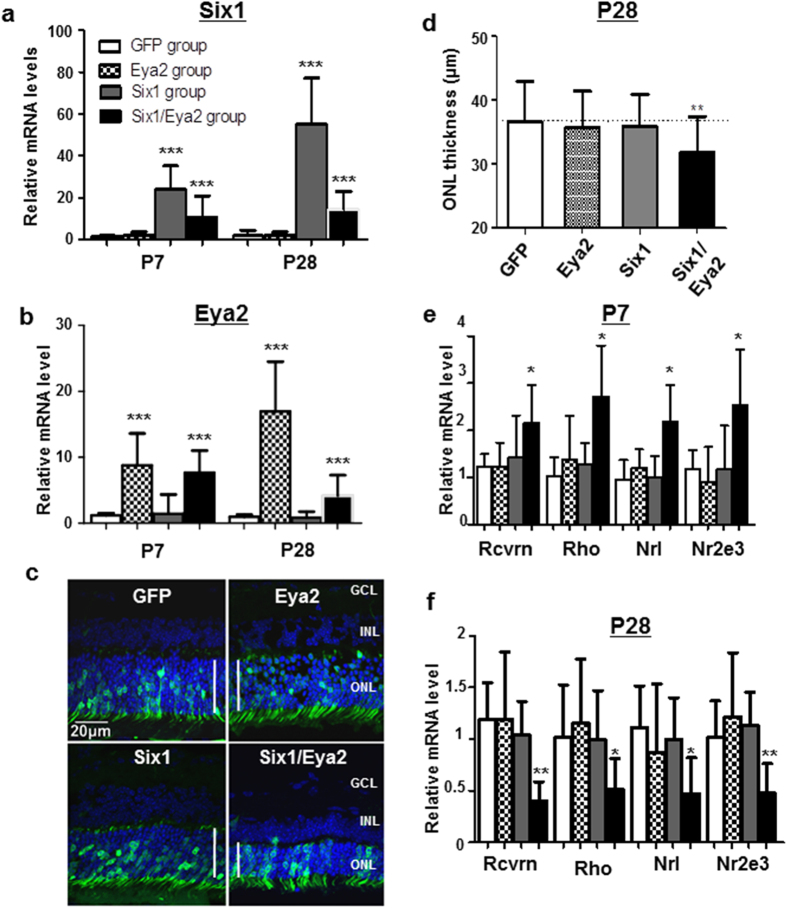
Forced expression of *Six1* and *Eya2* in postnatal retina induces photoreceptor degeneration. (**a,b**) qPCR analyses of *Six1* (**a**) and *Eya2* (**b**) mRNA levels in wild-type mouse retinae taken at P7 and P28 following P0 *in vivo* electroporation of *Six1*, *Eya2*, or *Six1* and *Eya2* simultaneously (*Six1/Eya2*) to the mouse retinae (n = 12/group). (**c**) Detection of *GFP* expression in 1 M mouse retinal sections after P0 *in vivo* electroporation of *GFP*, *Six1*, *Eya2*, or *Six1* and *Eya2* simultaneously. Note the robust detection of *GFP* expression in the ONL and the thinner ONL in *Six1/Eya2* electroporated retina compared to the other electroporated retina groups. Vertical lines mark the range of the ONL. (**d**) Quantification of ONL thicknesses in retinal sections of 1 M old mice that received *in vivo* electroporation of *GFP*, *Six1*, *Eya2*, or *Six1* and *Eya2* simultaneously at P0. (**e,f**) Results of qPCR showed increased mRNA levels of photoreceptor genes only in *Six1* and *Eya2* co-electroporated retinas at P7 (**e**) but reduced mRNA levels of photoreceptor genes at P28, when compared to the *GFP*-electroporated group. Bars represent the mean ± S.D. of at least six biological replicates. *Indicates *P* < 0.05, ** indicates *P* < 0.01.

The *Six* family is comprised of homeodomain-containing DNA-binding proteins which play crucial roles in *Drosophila* and vertebrate eye formation^21^,^22^. Association between aberrant *Six1* expression and photoreceptor degeneration has not been studied. We thus asked if upregulated *Six1* and *Eya2* in KO might phenocopy some aspects of photoreceptor degeneration seen in *Ezh2* KO retinae. We electroporated plasmids encoding *Six1, Eya*2 or *GFP* (as a control) into postnatal P0 WT retinae *in vivo*. When analyzed at 1 M, *GFP* was expressed in the ONL, and qRT-PCR confirmed elevated *Six1* and *Eya2* expression, respectively, in electroporated WT retinae (Fig. 6a–c). Dual *Six1*/*Eya2* electroporated retinae showed decreased ONL thickness at 1 M compared to controls (*GFP*), *Six1* or *Eya2* individually transfected groups (Fig. 6c,d). Associated with it, we noted decreased and increased levels of cell proliferation and apoptosis markers, *Ki67, Caspase-3*, respectively, in *Six1*/*Eya2*-electroporated group as compared to *GFP, Six1, or Eya2* individually-electroporated groups (Supplementary Fig. 6). Similar to *Ezh2* KO mice, *recoverin*, *rhodopsin*, *Nrl* and *Nr2e3* were up-regulated in *Six1/Eya2*-co-electroporated retinae at P7 as compared to control *GFP-*electroporated retinae (Fig. 6e), but they were down-regulated in the *Six1/Eya2* co-transfected group retina at P28 due to photoreceptor degeneration (Fig. 6f). The levels of *recoverin*, *rhodopsin*, *Nrl* and *Nr2e3* in *Six1* or *Eya2* individually transfected groups remained unchanged in both time points measured when were compared to control *GFP-*electroporated retinae. The data further support that *Six1*, which requires the presence of *Eya2,* directly mediates the expression of photoreceptor-related genes. Together, our data suggests that adult photoreceptor degeneration secondary to *Ezh2* inactivation during retinal development is mediated by aberrant expression of derepressed *Six1* and *Eya2*, which induces degeneration through decreased cell proliferation and increased apoptosis.

## Discussion

We have identified an epigenetic mechanism by which *Ezh2* expression during embryonic and early postnatal retinal development regulates photoreceptor homeostasis in the adult, a stage when *Ezh2* is not normally expressed. In the absence of *Ezh2*, *Six1* is aberrantly upregulated by *Ezh2*-mediated loss of H3K27me3 at its promoter. This causes misexpression of photoreceptor related genes that is, at least in part, contributing to progressive photoreceptor degeneration in this model. Our findings relate transiently expressed, developmental regulators to adult retinal neuron survival and function, and for the first time reveal an epigenetic link by which *in utero* events lead to juvenile and adult-onset vision loss.

Here we uncovered that, in addition to its role in stem cell self-renewal and lineage specification in development^23^, *Ezh2*-mediated gene repression orchestrates postnatal retinal neuron survival and function. During retinal development, a delicate balance between proliferation and differentiation of progenitors is essential for ensuring proper cell fate development and establishment of cell survival and function. Our data suggests that *Ezh2* governs this process through deposition of repressive modification H3K27me3 on early transcription factors, such as *Six1*, to allow stabilized expression of photoreceptor-related genes in the differentiated cell decedents^24^. The *Six* family of homeodomain-contain DNA-binding proteins is a group of transcription factors, which participate in eye formation during early development^25^. *Six1* acts together with its coactivators *Eya1* and *Eya2* to drive cell proliferation, tissue growth and cell fate specification in early and mid-eye development in *Drosophila*^26^. Yet, its involvement in mammalian retinal development has not been documented. Our results suggest that *Six1* is transiently expressed in the embryonic mouse retina and binds to the promoter regions of photoreceptor specific genes, including *rhodopsin* and *recoverin*, to mediate their levels of expression before birth. Overexpression of *Six1* in the postnatal stage results in destabilized photoreceptor gene expression in the mature retina and causes postnatal onset of retinal degeneration. These findings indicate that epigenetic dysregulation in retinal development is a predisposing factor for congenital photoreceptor dystrophy and postnatal photoreceptor degeneration. Future study investigating photoreceptor rescue in KO mice by deletion of *Six1* or *Eya2* (through crossing with *Six1* or *Eya2* deficient mice) may provide direct testimony if derepression of *Six1* is essential for photoreceptor degeneration following *Ezh2* loss-of-function.

Our findings suggest a previously unappreciated role of *Ezh2* in retinal homeostasis. Report by Aldiri *et al*.^27^ showed that in *Xenopus*, knockdown of *Ezh2* constrains the generation of retinal neurons and promotes a Müller glial cell fate, partially due to upregulation of Cdk inhibitor p15. In agreement with this finding, Iida *et al*.^13^ reported accelerated differentiation of Müller glia and rod photoreceptors, and impaired cell proliferation in *Dkk3-Cre* driven *Ezh2* deficient retinae. Using *Pax6*-α*Cre*, Zhang *et al*.^14^ observed similarly reduced proliferation of RPCs and diminished numbers of retinal ganglion cells and amacrine cells, which is accompanied by increased photoreceptor and Müller cell differentiation, following *Ezh2* deletion. Their RT-PCR and RNA sequencing results from the *Ezh2*-deficient retina revealed drastically upregulated *Six1*, in addition to other genes regulating early RPC development. Nevertheless, the developmental defects in their retinae prevented further elucidation of a role for *Ezh2* in retinal homeostasis. The difference between the phenotypes observed in the present study and those reported by others is presumably due to the fact that *Pax6*-α *Cre*^28^ and *Dkk3-Cre*^12^ induce Cre recombinase in early proliferating RPCs before E10.5; whereas, *Chx10-Cre* drives Cre expression in RPCs at a differentiation stage slightly later than that by *Pax6*-α *Cre* and *Dkk3-Cre*^16^. Thus, *Ezh2* deletion driven by *Chx10-Cre* results in gene expression changes that occur likely at the nexus point of cell proliferation and differentiation, persisting into adulthood, and lead to postnatal retinal degeneration.

It is intriguing that although highly expressed in RPCs and RGCs during perinatal period, *Ezh2* loss in all retinal progenitors (by use of the *Chx10-Cre* strain) selectively affects gene expression and homeostasis of photoreceptors, but not RGCs or other retinal cell types. This result is further confirmed by using *Math5-Cre*, which drives gene deletion from RPCs that are competent to generate RGCs^20^. The data reveal a unique and cell type specific regulation of gene expression by *Ezh2*, rather than genome-wide repression. While its underlying mechanisms remain uncertain, in part, this is unlikely to be caused solely by the spatiotemporal dynamics and cooperative functionalities of other HMTs and demethylases. Among the two HMTs that regulate H3K27 trimethylation, *Ezh1* and *Ezh2*, and the demethylase *Jmjd3*, *Ezh2* and *Jmjd3* are widely expressed in retinal progenitors during development; *Ezh1* is expressed only weakly in the postnatal retina^29^. The transient expression of *Ezh2* during development, which is completely repressed postnatally in the retina, was also confirmed during human retinal development^11^. A cell type specific effect was also observed with *Jmjd3* deficiency, which resulted in impaired differentiation of subsets of bipolar cells, despite strong *Jmjd3* expression in the RGCs^9^. Previous studies have reported a model of bidirectional and mutually reinforcing crosstalk between *Ezh2* and another repressive HMT, *G9a*, on histone hypermethylation in other tissues^30^,^31^. In support of a possible crosstalk between these two epigenetic silencing regulators in RGCs, *G9a* (which catalyzes H3K9me2, another repressive histone mark) is highly expressed in RGCs of the mouse retina during a similar period when *Ezh2* is detected^10^. It would be interesting to investigate the possible interactions of these repressive epigenetic modifications in RGCs such as by analyzing conditional knockouts of both *G9a* and *Ezh2* in the future.

In summary, our observations point to a central role for *Ezh2*-mediated histone methylation in the epigenetic repression of *Six1* and photoreceptor homeostasis in the adult. *Six1* and the targetable HMT epigenetic enzyme regulating *Six1, Ezh2,* thus may represent novel therapeutic targets in congenital photoreceptor degeneration.

## Methods

### Generation and genotyping of KO mice

All animal procedures were performed in accordance with the statement of the Association for Research in Vision and Ophthalmology, and the protocols were approved by Institutional Animal Care Committee (IACUC) of the Schepens Eye Research Institute. Mice were housed in a temperature- controlled room with a 12 h light/dark cycle. Fresh water and rodent diet were available at all times.

Mice carrying deletion of *Ezh2* specifically in all retinal progenitors was produced using the *Chx10-Cre* mice line (from the Jackson Laboratory) crossed with conditional allele of *Ezh2*^*flox/flox*^ mice (from the Mutant Mouse Regional Resource Centers (MMRRC) (https://www.mmrrc.org/catalog/sds.php?mmrrc_id=15499). *Chx10-cre; Ezh2*^*flox/flox*^ (KO) mice were eventually generated and allowed to investigate its function in retina development.

Mice were genotyped by PCR analysis. Mice were anesthetized with 2–4% isoflurane (Vedco Inc., MO, USA) and less than 0.5 cm of tail tissues were cut by razor blade. The tail tissue was incubated in direct PCR buffer solution (Viagen Biotech, CA, USA) containing proteinase K (Invitrogen Life Technologies, Carlsbad, CA, USA), and 56 °C overnight according to the manufacturer’s instruction. PCR genotyping was performed using the following primers: *Chx10-cre* forward primer 5′-GGGCACCTGGGACCAACTTCACGA-3′ and reverse primer 5′-CGGCGGCGGTCACGAACTCC-3′ (750 bp PCR product)^16^; *Ezh2* forward primer 5′-CTGCTCTGAATGGCAACTCC-3′ and reverse primer 5′-TTATTCATAGAGCCACCTGG-3′ (Control 430 bp and KO 470 bp). The PCR was performed using Apex Taq DNA Polymerase (Genesse Scientific, San Diego, CA). The primers were designed to detect the expression of *Ezh2* gene at P0 and 1 M. The primers information as follows: forward primer 5′-TCAGGATGAAGCAGACAGAAG-3′ and reverse primer 5′-TTTGTTGCCCTTTCGGGTTG-3′. The PCR reaction was carried out according to the manufacturer’s instruction.

### Hematoxylin and Eosin (HE) Staining and Immunohistochemistry (IHC)

Fixation, sectioning, HE staining were carried out as described^32^. For immunohistochemistry, mouse embryos and eyecups were fixed in 4% paraformaldehyde in PBS for either 30 min to 3 h. The samples were cryoprotected, embedded, frozen, and sectioned (8 μm thick). Slides were incubated with blocking solution (4% normal donkey serum and 0.5% Triton X-100 in PBS) for 1 h, and then with the primary antibodies overnight at 4 °C degree. Slides were washed with PBS three times for 10 min each time and incubated with the secondary antibodies for 1 h at room temperature. The specimens were observed under a laser confocal microscope (Leica TCS SP5).

Primary antibodies used in this study were *Beta-III Tubulin* (1:1000; AB9354, Millpore), *Recoverin* Antibody (1:1000; AB5585, Millpore), *PNA* Antibody (1:200; Sigma), *Ezh2* (1:1000; 3147s, cell signaling) and *H3K27me3* (1:500, 9733S, Cell Signaling). Second antibodies were *Cy3*-labeled donkey anti-mouse antibody (1:1500; Vector Laboratories), *Cy3*-labeled donkey anti-rabbit antibody (1:1500; Vector Laboratories) and Alexa Fluor 488 Conjugated antibody (1:400; cell signaling). For all quantifications, KO mice and controls were used for all analyses. Quantification of the thickness of nuclear staining for each antibody was carried out on fluorescence microscope images (Leica, Mannheim, Germany) with ImageJ software (National Institutes of Health, Bethesda, MD). Student’s *t* tests were used to measure differences between KO mice and controls.

### Transmission Electron Microscopy (TEM)

Mice were euthanized with CO_2_ and the eyes were enucleated and the posterior segments fixed in 1% glutaraldehyde/1% paraformaldehyde in PBS and postfixed in veronal acetate buffered osmium tetroxide (2%), dehydrated in ethanol and water, and embedded in Epon. Ultrathin sections were cut from blocks and mounted on copper grids. The specimens were examined by using a transmission electron microscope (Tecnai Spirit, FEI).

### Optical Coherence Tomography (OCT)

Retinal morphology and thickness of the ONL of live mice were assessed non-invasively with spectrum domain optical coherence tomography (SD-OCT). Mice were anesthetized with an intraperitoneal injection of ketamine (120 mg/kg)/xylazine (12 mg/kg) mixture and pupils were dilated using tropicamide (1%; Falcon Pharmaceuticals, Fort Worth, TX). Lubricant gel drops (Novartis Pharmaceuticals Corp, East Hanover, NJ) were applied to maintain the moisture of cornea. Images were acquired using SD-OCT (Bioptigen Inc, Research Triangle Park, NC). Radial volume scan (centered on optic disc, diameter 1.3 mm) was used, and ONL thickness within the 200–400 μm area from the central of optic disk were measured and analyzed for the ONL thickness as previously described^33^.

### Electroretinogram (ERG)

Retinal functions of control and KO mice were assessed by ERG. Mice were dark-adapted 5 h, and then anesthetized with intraperitoneal injection. Pupils were dilated with topical 1% tropicamide and 0.5% phenylephrine HCl, and the mice were placed on a heating pad for the duration of the ERG recordings. The mice were stimulated with stroboscopic stimuli with six levels of stimuli 0.00022, 0.00215, 0.06455, 0.64545, 2.15 and 500 cd-s/m^2^ to elicit the scotopic ERGs. After the scotopic measurement, mice were exposed to a rod-saturating background light (40 cd/m^2^) for 10 min, then the mice with stimuli 10 cd-s/m^2^ for the photopic ERGs. The data were recorded and processed by the ERG system (Espion Electroretinography System; Diagnosys LLC, Lowell, MA). The amplitudes of a-wave and b-wave of ERG response were determined.

### Microarray Expression Analysis

Total RNA was extracted from both cortices from three KO mice and controls at P0 stage. Total retinal RNA (5 μg) was isolated using RNeasy Plus Mini Kit (QIAGEN, Valencia, CA, USA) and converted to cDNA using the One-Cycle cDNA synthesis kit (Affymetrix, Santa Clara, CA, USA) according to the manufacturer’s instructions. Gene Expression of KO mice and controls were detected using the Affymetrix Mouse Gene 1.0 ST Array (Dana-Farber Cancer Institute) and the microarray data were analyzed using the dChip software (www.dchip.org). Gene Ontology (GO) analysis of genes up-regulated and down-regulated in the KO mice retina was performed using DAVID tools (http://david.abcc.ncifcrf.gov).

### Quantitative Real-Time PCR (qPCR)

Total retina RNA was extracted using RNeasy Plus Mini Kit (QIAGEN, Valencia, CA, USA) and cDNA synthase using SuperScript™ III First-Strand Synthesis System (Invitrogen Life Technologies, Carlsbad, CA, USA). QPCR was performed using SYBR FAST qPCR Kits (Kapa Biosystems, Woburn, MA, USA) and LightCycler^®^ 480 (Roche Biosystems; Indianapolis, IN) according to the manufacturer’s instructions. The primer sequences are listed in Supplementary Table 1. For relative comparison of each gene, we analyzed the *Ct* value of real-time PCR data with the ∆∆Ct method normalizing by an endogenous control (glyceraldehyde 3-phosphate dehydrogenase, *GAPDH*).

### Chromatin immunoprecipitation (ChIP)

The P0 retina tissues of KO mice and controls were cut into small pieces (between 1–3 mm^3^) and the tissues were cross-linked with 1% paraformaldehyde (#252549, Sigma) in PBS for 1 hour at 37 °C. Then chromatin isolation and ChIP assay were performed according to the manufacturer’s protocol using a commercially available kit (EZ-Zyme Chromatin prep kit, EZ-ChIP; Millipore). The immunoprecipitated chromatin DNA was incubated with rabbit anti-*H3K27me3* antibody (#9756s, Cell Signaling) and goat anti-*six1* antibody (Santa Cruz) and was analyzed by qPCR using gene-specific primers (Supplementary Table 1).

### RGC qualification

The mouse retinas were dissected to remove the cornea, lens, iris, sclera, and choroid. The vitreous was carefully removed and the flat mounted retina was placed on the super-frost sections with ganglion cell layer facing up. RGCs were double immunolabeled with *β-III tubulin* (Tuj1) and DAPI. Our previous studies showed that Tuj1 specifically labels RGCs in retinal flat-mounts^34^. The specimens were visualized under the confocal microscope at 800× magnification. The flat mounts were divided into quadrants: superior, temporal, nasal and inferior and total 16 square regions of each eye were photographed and all *β-III tubulin* positive cells in the GCL were quantified. The average RGC densities of the whole retina were calculated and the percentage of RGC loss was determined by comparing RGC densities with that obtained from the contralateral control eyes.

### *In vivo* Electroporation

Three of the expression vectors were purchased from GeneCopoeia (Germantown, MD, USA), including *pEZ-M90* (*GFP* report), *pEZ-M90-Six1* and *pEZ-M90-Eya2*. The experiment was divided into four groups: *pEZ-M90* (*GFP* group), *pEZ-M90-Six1* (*Six1* group), *pEZ-M90-Eya2* (*Ezh2* group), and *pEZ-M90-Six1* mixed with *pEZ-M90-Eya2* (*Six1/Ezh2* group). The plasmids were amplified using QIAGEN Plasmid Maxi Kit (Valencia, CA, USA). Newborn ICR mouse pups were anesthetized by chilling on ice, and a small incision was made in the eyelid and sclera near the lens with a 32-gauge needle. 0.5–1 μl DNA solutions (1 μg/μl) in PBS containing 0.1% blue dye as a tracer were injected into the subretinal space through the incision by using a Hamilton syringe (Reno, Nevada, USA) with a 32 gauge blunt-ended needle under a dissecting microscope. After DNA injection, 10 mm diameter tweezer-type electrodes (Nepagene, Chiba, Japan) briefly soaked in PBS were placed to softly hold the heads of the pups. Five square pulses of 80 volts and 50 ms duration with 950 ms intervals were applied by using a NEPA21 Electroporator (Nepagene, Chiba, Japan).

The eyeballs were harvested at 7 and 28 days, respectively, after electroporation. The eyeballs were fixed with 4% paraformaldehyde, cryoprotected with 30% sucrose, embedded in OCT compound (Sakura Finetek, Tokyo, Japan) and cryosectioned into 8 μm section. The *GFP*-positive retina sections were selected under a Leica DM3000 fluorescent microscope. Only retinal sections containing 1/3 or more GFP expressing cells were selected for scoring. Mouse eyes that developed cataract, small eyes, or apparent inflammation were excluded. For each group, at least 12 *GFP-*positive retinae were scored. Quantification of the thickness of ONL for each group was carried out on fluorescence microscope images with NIH ImageJ software. At 7 and 28 days after electroporation, electroporated retinae were dissected under a fluorescent microscope to select the *GFP*-positive retinae. Total retina RNA was extracted and cDNA was synthesized. QPCR was performed and the results were analyzed with the ∆Ct method.

### Statistical Analysis

The statistical significance of IHC, OCT, ERG and qPCR results were analyzed by using using GraphPad Prism 5 statistical software (GraphPad Software, La Jolla, CA). All the numerical variables in this article were presented as Mean ± S.E.M. Two-tailed student’s *t*-test was used on numerical variables of independent samples; one-way ANOVA analysis was applied for comparisons of data among groups.

## Additional Information

**How to cite this article**: Yan, N. *et al*. Postnatal onset of retinal degeneration by loss of embryonic *Ezh2* repression of Six1. *Sci. Rep.* **6**, 33887; doi: 10.1038/srep33887 (2016).

## Supplementary Material

Supplementary Information

## Figures and Tables

**Table 1:** Representative up and down-regulated genes in Chx10-cre Ezh2^*flox/flox*^ mice P0 retina from microarray hybridization.

NM_175540	Eda2r	ectodysplasin A2 isoform receptor	3.95
NM_009189	Six1	sine oculis-related homeobox 1 homolog (Drosophila)	3.83
NM_009849	Entpd2	ectonucleoside triphosphate diphosphohydrolase 2	3.19
NM_008992	Abcd4	ATP-binding cassette, sub-family D (ALD), member 4	2.93
NM_007723	Cnga1	cyclic nucleotide gated channel alpha 1	2.41
NM_028864	Zc3hav1	zinc finger CCCH type, antiviral 1	2.32
NM_013750	Phlda3	pleckstrin homology-like domain, family A, member 3	2.31
NM_030707	Fcrls	Fc receptor-like S, scavenger receptor	2.3
NM_013708	Nr2e3	nuclear receptor subfamily 2, group E, member 3	2.29
NM_023048	Asb4	ankyrin repeat and SOCS box-containing 4	2.27
NM_182993	Slc17a7	solute carrier family 17 (sodium-dependent inorganic phosphate cotransporter), member 7	2.25
NM_145383	Rho	rhodopsin	2.23
NM_011978	Slc27a2	solute carrier family 27 (fatty acid transporter), member 2	2.2
NM_009038	Rcvrn	recoverin	2.19
NM_009022	Aldh1a2	aldehyde dehydrogenase family 1, subfamily A2	2.18
NM_008806	Pde6b	phosphodiesterase 6B, cGMP, rod receptor, beta polypeptide	2.1
BC086653	Lba1	lupus brain antigen 1	2
NM_010720	Lipg	lipase, endothelial	1.89
NM_010165	Eya2	eyes absent 2 homolog (Drosophila)	1.88
NM_011851	Nt5e	5′ nucleotidase, ecto	1.88
NM_001031772	Lin28b	lin-28 homolog B (C. elegans)	1.85
NM_023456	Npy	neuropeptide Y	1.85
NM_008630	Mt2	metallothionein 2	1.82
NM_007669	Cdkn1a	cyclin-dependent kinase inhibitor 1A (P21)	1.81
NM_133982	Rpp25	ribonuclease P 25 subunit (human)	1.79
NM_176844	Chrna5	cholinergic receptor, nicotinic, alpha polypeptide 5	1.78
NM_008736	Nrl	neural retina leucine zipper gene	1.78
NM_028713	Rftn2	raftlin family member 2	1.76
NM_013602	Mt1	metallothionein 1	1.73
NM_001033167	Slc22a23	solute carrier family 22, member 23	1.73
NM_001128103	Ano3	anoctamin 3	1.72
NM_031257	Plekha2	pleckstrin homology domain-containing, family A (phosphoinositide binding specific) member 2	1.7
NM_009573	Zic1	zinc finger protein of the cerebellum 1	1.7
NM_008532	Tacstd1	tumor-associated calcium signal transducer 1	1.66
NM_178254	Tcfl5	transcription factor-like 5 (basic helix-loop-helix)	1.66
NM_013454	Abca1	ATP-binding cassette, sub-family A (ABC1), member 1	1.64
NM_019397	Egfl6	EGF-like-domain, multiple 6	1.63
NM_027871	Arhgef3	Rho guanine nucleotide exchange factor (GEF) 3	1.62
NM_172119	Dio3	deiodinase, iodothyronine type III	1.62
NM_019413	Robo1	roundabout homolog 1 (Drosophila)	1.58
NM_153546	Mboat1	membrane bound O-acyltransferase domain containing 1	−1.57
NM_009657	Aldoc	aldolase C, fructose-bisphosphate	−1.62
NM_172880	Tmprss11e	transmembrane protease, serine 11e	−1.65
NM_008397	Itga6	integrin alpha 6	−1.66
NM_009528	Wnt7b	wingless-related MMTV integration site 7B	−1.66
NM_033268	Actn2	actinin alpha 2	−1.68
NM_025681	Lix1	limb expression 1 homolog (chicken)	−1.69
NM_007515	Slc7a3	solute carrier family 7 (cationic amino acid transporter, y+ system), member 3	−1.7
NM_015800	Crim1	cysteine rich transmembrane BMP regulator 1 (chordin like)	−1.71
NM_001113331	Shc1	src homology 2 domain-containing transforming protein C1	−1.77
NM_011990	Slc7a11	solute carrier family 7 (cationic amino acid transporter, y+ system), member 11	−1.77
NM_011419	Jarid1d	jumonji, AT rich interactive domain 1D (Rbp2 like)	−1.79
NM_173379	Leprel1	leprecan-like 1	−1.81
NM_009932	Col4a2	collagen, type IV, alpha 2	−1.82
NM_001035533	Akap2	A kinase (PRKA) anchor protein 2	−1.83
NM_012008	Ddx3y	DEAD (Asp-Glu-Ala-Asp) box polypeptide 3, Y-linked	−1.84
NM_009527	Wnt7a	wingless-related MMTV integration site 7A	−1.84
NM_027934	Rnf180	ring finger protein 180	−1.88
NM_013737	Pla2g7	phospholipase A2, group VII (platelet-activating factor acetylhydrolase, plasma)	−1.89
NM_007514	Slc7a2	solute carrier family 7 (cationic amino acid transporter, y+ system), member 2	−1.89
NM_009484	Uty	ubiquitously transcribed tetratricopeptide repeat gene, Y chromosome	−1.9
NM_012011	Eif2s3y	eukaryotic translation initiation factor 2, subunit 3, structural gene Y-linked	−1.95
NM_025760	Ptplad2	protein tyrosine phosphatase-like A domain containing 2	−1.97
NM_172838	Slc16a12	solute carrier family 16 (monocarboxylic acid transporters), member 12	−1.97
NM_009848	Entpd1	ectonucleoside triphosphate diphosphohydrolase 1	−2.03
NM_007933	Eno3	enolase 3, beta muscle	−2.05
NM_026878	Rasl11b	RAS-like, family 11, member B	−2.11
NM_025769	Efcab1	EF hand calcium binding domain 1	−2.18
NM_146120	Gsn	gelsolin	−2.23
NM_198191	Pip5kl1	phosphatidylinositol-4-phosphate 5-kinase-like 1	−2.24
NM_026056	Cap2	CAP, adenylate cyclase-associated protein, 2 (yeast)	−2.25
NM_172868	Palm2	paralemmin 2	−2.25
NM_007773	Crybb2	crystallin, beta B2	−2.26
NM_011939	Hsf4	heat shock transcription factor 4	−2.28
NM_172152	Slc24a4	solute carrier family 24 (sodium/potassium/calcium exchanger), member 4	−2.3
NM_144945	Lgi2	leucine-rich repeat LGI family, member 2	−2.34
ENSMUST00000108875	Birc7	baculoviral IAP repeat-containing 7 (livin)	−2.35
NM_177780	Dock5	dedicator of cytokinesis 5	−2.38
NM_008180	Gss	glutathione synthetase	−2.4
NM_008010	Fgfr3	fibroblast growth factor receptor 3	−2.43
NM_011224	Pygm	muscle glycogen phosphorylase	−2.43
NM_025711	Aspn	asporin	−2.49
NM_013822	Jag1	jagged 1	−2.68
NM_146142	Tdrd7	tudor domain containing 7	−2.73
NM_030127	Htra3	HtrA serine peptidase 3	−2.84
NM_177041	Flad1	RFad1, flavin adenine dinucleotide synthetase, homolog (yeast)	−2.9
NM_181541	Caprin2	caprin family member 2	−3.1
NM_001113368	Ceacam2	carcinoembryonic antigen-related cell adhesion molecule 2	−3.12
NM_011325	Scnn1b	sodium channel, nonvoltage-gated 1 beta	−3.14
NM_007776	Crygd	crystallin, gamma D	−3.19
NM_026439	Ccdc80	coiled-coil domain containing 80	−3.34
NM_138953	Ell2	elongation factor RNA polymerase II 2	−3.34
XR_032001	Csnk2a1-rs2	casein kinase 2, alpha 1, related sequence 2	−3.39
NM_010442	Hmox1	heme oxygenase (decycling) 1	−3.68
NM_028813	Vit	vitrin	−3.74
NM_001011807	Olfr191	olfactory receptor 191	−4
NM_019738	Nupr1	nuclear protein 1	−4.03
NM_138683	Rspo1	R-spondin homolog (Xenopus laevis)	−4.23
NM_008100	Gcg	glucagon	−4.64
NM_010917	Nid1	nidogen 1	−4.7
NM_013501	Cryaa	crystallin, alpha A	−4.79
NM_009604	Chrng	cholinergic receptor, nicotinic, gamma polypeptide	−5.11
NM_146405	Olfr228	olfactory receptor 228	−5.4
NM_145835	Lctl	lactase-like	−5.46
NM_018870	Pgam2	phosphoglycerate mutase 2	−5.51
NM_175013	Pgm5	phosphoglucomutase 5	−5.55
NM_027010	Crygf	crystallin, gamma F	−5.58
NM_144805	Tmem40	transmembrane protein 40	−5.68
NM_008048	Igfbp7	insulin-like growth factor binding protein 7	−6.14
NM_020288	Olfr749	olfactory receptor 749	−6.33
NM_030022	Grifin	galectin-related inter-fiber protein	−7.11
NM_023695	Crybb1	crystallin, beta B1	−7.18
NM_008760	Ogn	osteoglycin	−7.83
NM_009965	Cryba1	crystallin, beta A1	−8.96
NM_007774	Cryga	crystallin, gamma A	−9.34
NM_019957	Dnase2b	deoxyribonuclease II beta	−9.61
NM_021352	Crybb3	crystallin, beta B3	−9.66
NM_007777	Cryge	crystallin, gamma E	−10.13
NM_009964	Cryab	crystallin, alpha B	−11.17
NM_153076	Crygn	crystallin, gamma N	−12.64
NM_007601	Capn3	calpain 3	−13.48
NM_144761	Crygb	crystallin, gamma B	−13.48
NM_008123	Gja8	gap junction protein, alpha 8	−14.36
NM_001002896	Bfsp2	beaded filament structural protein 2, phakinin	−15.1
NM_009751	Bfsp1	beaded filament structural protein 1, in lens-CP94	−15.4
NM_021541	Cryba2	crystallin, beta A2	−17.03
NM_016975	Gja3	gap junction protein, alpha 3	−17.29
NM_001082573	Crygc	crystallin, gamma C	−17.63
NM_021351	Cryba4	crystallin, beta A4	−21.81
NM_008600	Mip	major intrinsic protein of eye lens fiber	−21.81
NM_177693	Lim2	lens intrinsic membrane protein 2	−23.85
NM_009967	Crygs	crystallin, gamma S	−25.42

## References

[b1] Michaelides M., Hardcastle A. J., Hunt D. M. & Moore A. T. Progressive cone and cone-rod dystrophies: phenotypes and underlying molecular genetic basis. Surv Ophthalmol 51, 232–258, 10.1016/j.survophthal.2006.02.007 (2006). 16644365

[b2] Moore A. T. Childhood macular dystrophies. Curr Opin Ophthalmol 20, 363–368, 10.1097/ICU.0b013e32832f8002 (2009). 19587597

[b3] Nakamura M., Lin J. & Miyake Y. Young monozygotic twin sisters with fundus albipunctatus and cone dystrophy. Arch Ophthalmol 122, 1203–1207, 10.1001/archopht.122.8.1203 (2004). 15302662

[b4] Berghmans L. V. . Discordance for retinitis pigmentosa in two monozygotic twin pairs. Retina 31, 1164–1169, 10.1097/IAE.0b013e3181fbcf2b (2011). 21283054

[b5] Walia S. . Discordant phenotypes in fraternal twins having an identical mutation in exon ORF15 of the RPGR gene. Arch Ophthalmol 126, 379–384, 10.1001/archophthalmol.2007.72 (2008). 18332319

[b6] Gluckman P. D., Hanson M. A., Cooper C. & Thornburg K. L. Effect of in utero and early-life conditions on adult health and disease. N Engl J Med 359, 61–73, 10.1056/NEJMra0708473 (2008). 18596274 PMC3923653

[b7] Cao R. & Zhang Y. The functions of E(Z)/EZH2-mediated methylation of lysine 27 in histone H3. Curr Opin Genet Dev 14, 155–164, 10.1016/j.gde.2004.02.001 (2004). 15196462

[b8] Pereira J. D. . Ezh2, the histone methyltransferase of PRC2, regulates the balance between self-renewal and differentiation in the cerebral cortex. Proceedings of the National Academy of Sciences of the United States of America 107, 15957–15962, 10.1073/pnas.1002530107 (2010). 20798045 PMC2936600

[b9] Iida A. . Histone demethylase Jmjd3 is required for the development of subsets of retinal bipolar cells. Proc Natl Acad Sci USA 111, 3751–3756, 10.1073/pnas.1311480111 (2014). 24572572 PMC3956141

[b10] Rao R. C. . Dynamic patterns of histone lysine methylation in the developing retina. Invest Ophthalmol Vis Sci 51, 6784–6792, 10.1167/iovs.09-4730 (2010). 20671280 PMC3055777

[b11] Khan M. . Characterization and pharmacologic targeting of EZH2, a fetal retinal protein and epigenetic regulator, in human retinoblastoma. Lab Invest 95, 1278–1290, 10.1038/labinvest.2015.104 (2015). 26280220 PMC4626270

[b12] Sato S. . Dkk3-Cre BAC transgenic mouse line: a tool for highly efficient gene deletion in retinal progenitor cells. Genesis 45, 502–507, 10.1002/dvg.20318 (2007). 17661397

[b13] Iida A. . Roles of histone H3K27 trimethylase Ezh2 in retinal proliferation and differentiation. Developmental neurobiology 75, 947–960, 10.1002/dneu.22261 (2015). 25556712

[b14] Zhang J. . Ezh2 maintains retinal progenitor proliferation, transcriptional integrity, and the timing of late differentiation. Dev Biol 403, 128–138, 10.1016/j.ydbio.2015.05.010 (2015). 25989023 PMC4469612

[b15] Dhomen N. S. . Absence of chx10 causes neural progenitors to persist in the adult retina. Invest Ophthalmol Vis Sci 47, 386–396, 10.1167/iovs.05-0428 (2006). 16384989 PMC2423807

[b16] Rowan S. & Cepko C. L. Genetic analysis of the homeodomain transcription factor Chx10 in the retina using a novel multifunctional BAC transgenic mouse reporter. Dev Biol 271, 388–402, 10.1016/j.ydbio.2004.03.039 (2004). 15223342

[b17] Liang L. & Sandell J. H. Focus on molecules: homeobox protein Chx10. Experimental eye research 86, 541–542, 10.1016/j.exer.2007.03.004 (2008). 17582398

[b18] Huang D. W. . DAVID Bioinformatics Resources: expanded annotation database and novel algorithms to better extract biology from large gene lists. Nucleic acids research 35, W169–W175, 10.1093/nar/gkm415 (2007). 17576678 PMC1933169

[b19] Huang da W., Sherman B. T. & Lempicki R. A. Systematic and integrative analysis of large gene lists using DAVID bioinformatics resources. Nature protocols 4, 44–57, 10.1038/nprot.2008.211 (2009). 19131956

[b20] Yang Z., Ding K., Pan L., Deng M. & Gan L. Math5 determines the competence state of retinal ganglion cell progenitors. Dev Biol 264, 240–254 (2003). 14623245 10.1016/j.ydbio.2003.08.005

[b21] Oliver G. & Gruss P. Current views on eye development. Trends Neurosci 20, 415–421, S0166-2236(97)01082-5 (1997). 9292971 10.1016/s0166-2236(97)01082-5

[b22] Kumar J. P. The sine oculis homeobox (SIX) family of transcription factors as regulators of development and disease. Cell Mol Life Sci 66, 565–583, 10.1007/s00018-008-8335-4 (2009). 18989625 PMC2716997

[b23] Conerly M. L., MacQuarrie K. L., Fong A. P., Yao Z. & Tapscott S. J. Polycomb-mediated repression during terminal differentiation: what don’t you want to be when you grow up? Genes Dev 25, 997–1003, 10.1101/gad.2054311 (2011). 21576260 PMC3093125

[b24] Margueron R. & Reinberg D. The Polycomb complex PRC2 and its mark in life. Nature 469, 343–349, 10.1038/nature09784 (2011). 21248841 PMC3760771

[b25] Anderson A. M., Weasner B. M., Weasner B. P. & Kumar J. P. Dual transcriptional activities of SIX proteins define their roles in normal and ectopic eye development. Development 139, 991–1000; 10.1242/dev.077255 (2012). 22318629 PMC3274360

[b26] Heanue T. A. . Synergistic regulation of vertebrate muscle development by Dach2, Eya2, and Six1, homologs of genes required for Drosophila eye formation. Genes Dev 13, 3231–3243 (1999). 10617572 10.1101/gad.13.24.3231 PMC317207

[b27] Aldiri I., Moore K. B., Hutcheson D. A., Zhang J. & Vetter M. L. Polycomb repressive complex PRC2 regulates Xenopus retina development downstream of Wnt/beta-catenin signaling. Development 140, 2867–2878, 10.1242/dev.088096 (2013). 23739135 PMC3699278

[b28] Marquardt T. . Pax6 is required for the multipotent state of retinal progenitor cells. Cell 105, 43–55 (2001). 11301001 10.1016/s0092-8674(01)00295-1

[b29] Watanabe S. & Murakami A. Regulation of Retinal Development via the Epigenetic Modification of Histone H3. Adv Exp Med Biol 854, 635–641, 10.1007/978-3-319-17121-0_84 (2016). 26427469

[b30] Coward W. R., Feghali-Bostwick C. A., Jenkins G., Knox A. J. & Pang L. A central role for G9a and EZH2 in the epigenetic silencing of cyclooxygenase-2 in idiopathic pulmonary fibrosis. FASEB J 28, 3183–3196, 10.1096/fj.13-241760 (2014). 24652950 PMC4062820

[b31] Mozzetta C. . The histone H3 lysine 9 methyltransferases G9a and GLP regulate polycomb repressive complex 2-mediated gene silencing. Mol Cell 53, 277–289, 10.1016/j.molcel.2013.12.005 (2014). 24389103

[b32] Fischer A. H., Jacobson K. A., Rose J. & Zeller R. Hematoxylin and eosin staining of tissue and cell sections. CSH Protoc 2008, pdb prot4986, 10.1101/pdb.prot4986 (2008). 21356829

[b33] Yang Q. . Microbead-induced ocular hypertensive mouse model for screening and testing of aqueous production suppressants for glaucoma. Invest Ophthalmol Vis Sci 53, 3733–3741, 10.1167/iovs.12-9814 (2012). 22599582 PMC3390181

[b34] Chen H. . Optic neuropathy due to microbead-induced elevated intraocular pressure in the mouse. Invest Ophthalmol Vis Sci 52, 36–44, 10.1167/iovs.09-5115 (2011). 20702815 PMC3053285

