# Supplementary material for: Postnatal onset of retinal degeneration by loss of embryonic Ezh2 repression of Six1
Source: Sci Rep. 2016 Sep 28;6:33887. doi: 10.1038/srep33887 (PMC5039414; doi:10.1038/srep33887)
Supplement: Supplementary Information [file srep33887-s1.pdf]

Supplementary Materials

Postnatal onset of retinal degeneration by loss of embryonic *Ezh2* repression of Six1

Naihong Yan[1,2], Lin Cheng[2,3], Kinsang Cho[2], Muhammad Taimur A. Malik[2], Lirong Xiao[1], Chenying Guo[2], Honghua Yu[2], Ruilin Zhu[2], Rajesh C. Rao[2,4,5,6,7], Dong Feng Chen[2,8]

Supplementary Figures

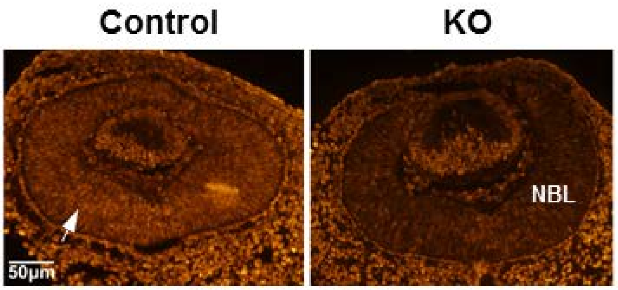

Supplementary Figure 1: Decreased *H3K27me3* deposition (red) in E13.5 *KO* retina. Retinal sections of E13.5 KO and littermate control mice were stained for H3K27me3. The white arrowhead points to H3K27me3 deposition in E13.5 control mouse retina. NBL: neuroblast layer.

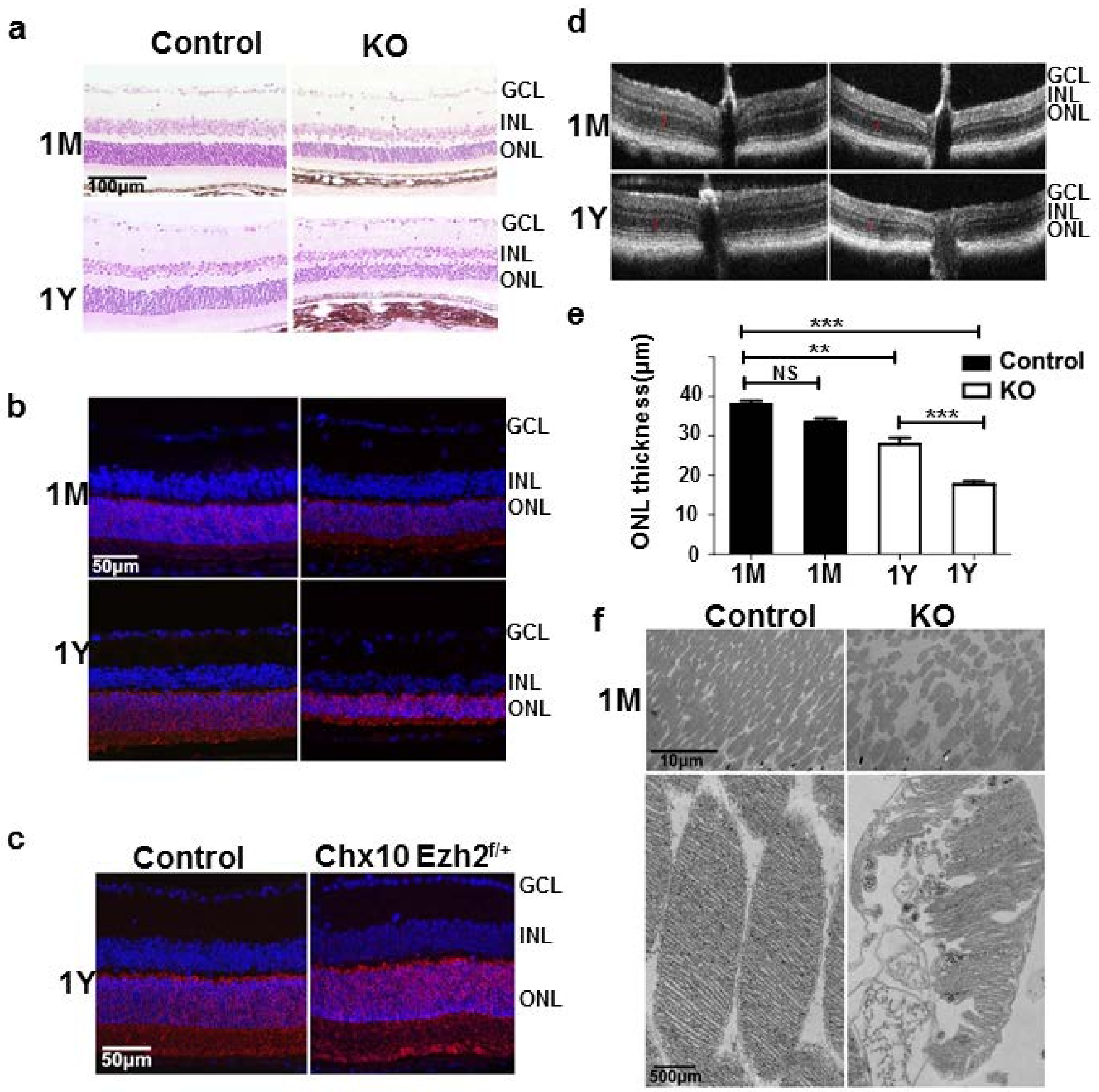

Supplementary Figure 2: *Ezh2* deletion leads to morphological and ultrastructural retinal changes. (a) H&E staining of whole retina sections revealed reduced ONL thickness in KO mice at 1M and 1Y. (b) Fewer *recoverin*$^{+}$ (red) photoreceptors are noted in KO mice at 1M and 1Y. DAPI is blue. (c) The pattern of recoverin staining and retinal layer thickness was the same between *Chx10-cre Ezh2*$^{flox/+}$ and control (*Ezh2*$^{flox/flox}$) mice at 1Y. (d,e) *In vivo* OCT retinal imaging showed reduced ONL thickness (red bar). (f) Ultrastructure of photoreceptor outer segments (OS) by electron microscopy showed well-aligned OS discs in control retinae but KO OS discs were fewer, poorly compacted and disorganized. Abbreviations: ganglion cell layer (GCL), inner plexiform layer (IPL), inner nuclear layer (INL), outer plexiform layer (OPL) and outer nuclear layer (ONL). Bars represent the mean ± S.D. of at least six biological replicates. *indicates P < 0.05, ** indicates P < 0.01, *** indicates P < 0.001.

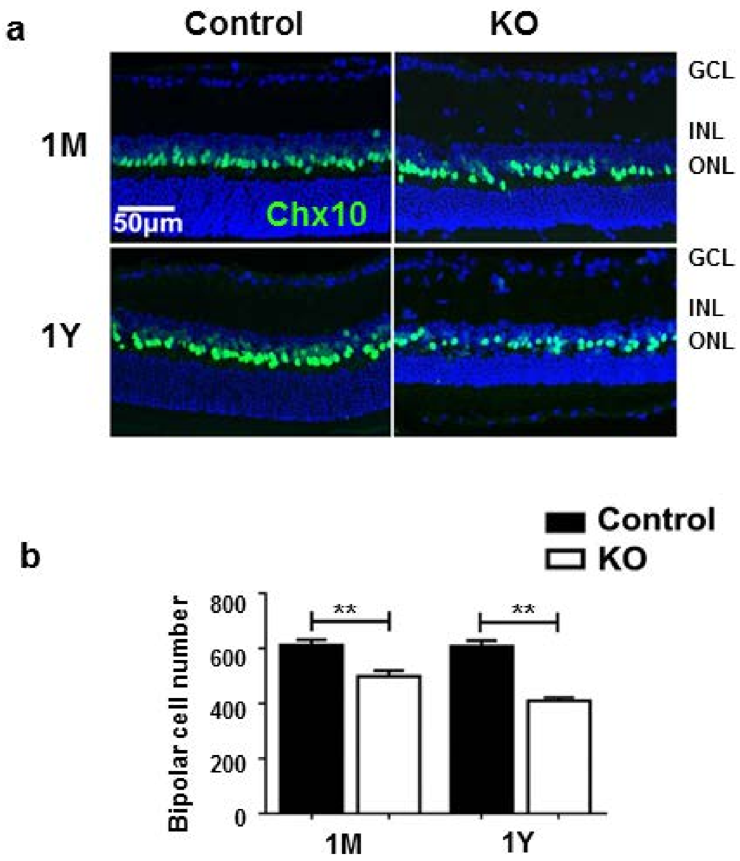

Supplementary Figure 3: *Ezh2* deletion results in postnatal cell type specific degeneration. KO retinae displayed fewer bipolar cells (Chx10, green) at 1M and 1Y compared to the littermate controls. Bars represent the mean ± S.D. of at least six biological replicates. ** indicates $P < 0.01$.

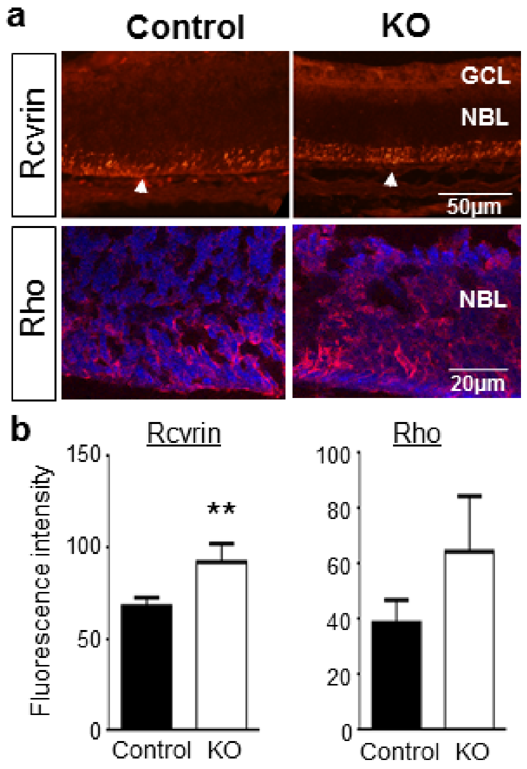

Supplementary Figure 4: Moderate photoreceptor gene expression in the KO retinae. (a) Retinal sections of P3 control and KO mice double-labeled with DAPI (blue; to reveal retinal structure) and primary antibody against either Recoverin (red; Rcvrin) or Rhodopsin (red; Rho) showing slightly increased immunofluorescent intensity in the KO retina vs control retina. (b) Image J quantification of fluorescence intensity for Recoverin (Rcvrin) and Rhodopsin (Rho) immunolabeling also revealed moderately increased fluorescence intensity in KO vs control retinae. Bars represent the mean ± S.D. of biological triplicates.

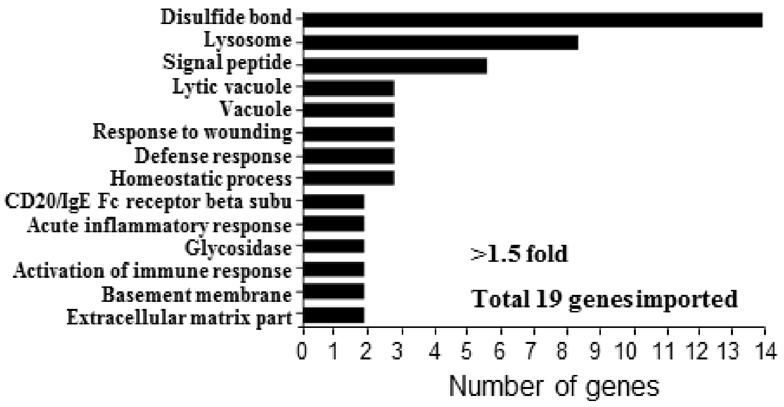

Supplementary Figure 5: Gene Ontology analysis of microarray data from Math5-KO RGCs.

Go analysis identified functional categories of genes associated with general cellular functions, such as lysosome and signal peptide, but none were specifically related to RGCs or RPCs. Bars represent the mean ± S.D. of at least eight biological replicates.

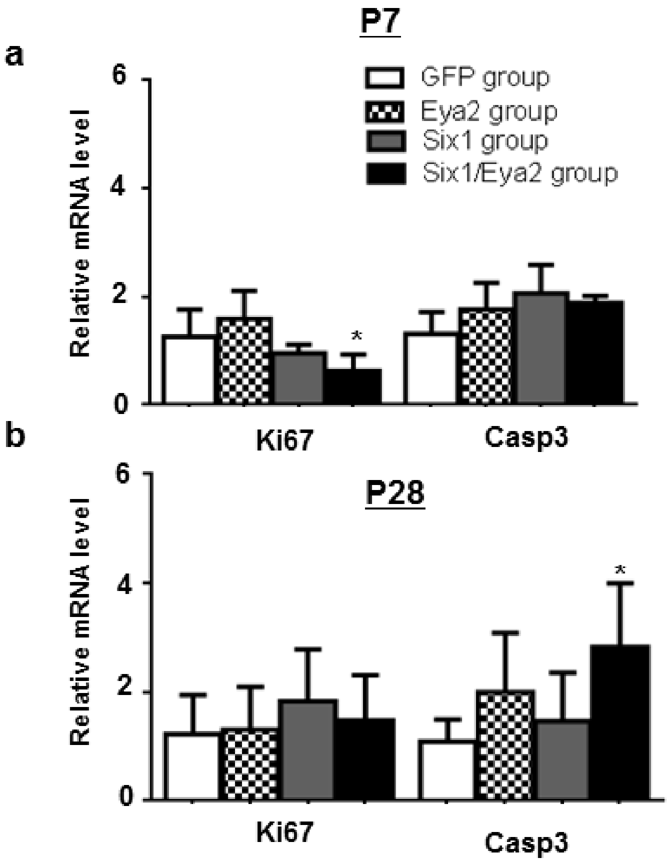

Supplementary Figure 6: Increasing levels of cell proliferation and apoptosis markers. Quantification of cell proliferation marker Ki67 and cell apoptosis marker caspase-3 in retinae received electroporation of *GFP*, *Six1*, *Eya2*, and *Six1+Eya2* plasmid with qPCR. A deceased level of Ki67 expression was detected only in *Six1+Eya2* electrophorated retina at 7days post treatment, while the increased level of expression of caspase-3 was found in *Six1+Eya2* electrophorated retina at 28 days post electroporation. Bars represent the mean ± S.D. of at least eight biological replicates. *indicates $P < 0.05$.

Supplementary Table 1. Primer sequences

	Gene	Forward	Reverse
Q-PCR	Sox2	AGAACCCCAAGATGCACAAC	CTCCGGGAAGCGTGTACTTA
	Pax6	AACAACCTGCCTATGCAACC	ACTTGGACGGGAACTGACAC
	Chx10	TTCAATGAAGCCCACTACCC	ATCCTTGGCAGACTTGAGGA
	Math5	CAGGACAAGAAGCTGTCCAA	CATAGGGCTCAGGGTCTACCT
	GAPDH	AACTTTGGCATTGTGGAAGG	ACACATTGGGGGTAGGAACA
	Recoverin	GCAGCTTCGATGCCAACAG	TCATGTGCAGAGCAATCAGGTA
	Pkca	CGCGAGGACAGCCTGTCT	AGAACCCTTCAAAATCAGATTGGT
	Rhodopsin	CATGCCAATATGCCCACCTT	GCACTGTGTTTCTGAACTCTTCAGA
	Cralbp	CTGTCCAGGGTGGAGGTCAT	CCCCAGCACCAAGGATCAC
	Crx	TCTGTGTGTTACAGACATGACCACTAA	CATCAAGCTTCTTTTGCATTTTGT
	NgN2	TCAGAGCTGCTGGAGGAGAAC	CCAGTTGCATTCCCTCTGAGA
	Nr2e3	CTTCATGGCTGTCAAATGG	CAGGCAAACTCTGTGGGAT
	Opsin 1	CCTGGCTACTTGGATTATTGG	CTGGGAGTAGGAGAAGCAGAT
	Blimp1	AGCATGACCTGACATTGACACC	CTCAACACTCTCATGTAAGAGGC
	Tuj1	CCAAGTTCTGGGAGGTCATC	TGAGAGGAGGCCTCATTGTAG
	Brn3a	CTCACGCTCTCGCACAAC	AGAGCTCCGGCTTGTTCAT
	Nrl	A A C T T CTGAGCATCGTGGCA	TGAAGAGTCGTGACCTGCAAA
	Six1	CTTGTACATAGAAGCCAGGGACAA	AGGGACTACTGTAAAGGATGCC
	Nupr1	CTAGAGGATGAAGATGGAATCCTG	TGGTGTCTGTGGTCTGGCCTTAT
	Eya1	CGTCCACCAATGCCACTTAC	GTGGAAAACAATGATGGTCTCGT
	Eya2	CTCCCTGAAAGCCCTCAATC	TGTCTTGGTCGCACTGTAGATG
	Ki67	GAGTGAGAGGGCAGTTCTGG	GCTGCAGTAACTGTGGGTCA
	Caspase-3	CTGTACGCGCACAAGCTAGA	CTTTGCGTGGAAAGTGGAGT
ChIP	Six1	CGAGGTTCGACTGGTCTCTTC	GCGCGGCTGCTCCTAA
	Recoverin	CTCCTCCCTCCAAGGACTG	CAAGGCTGTGTGCTGCTATG
	Rhodopsin	CCCCTCTGCAAGCCAATT	GCAACTCCAGGCACTGAC
	Nrl	CCTCGAACTCAGAAATCCGC	GTGTAGGAGTTGGGCAGGGT
	Nr2e3	CTCCTAACTTACAAGGCAAGGGA	CCTGCTTGCTAGAAGTTGCTGG
